# The development of cognitive and affective empathy from ages 3 to 20: a new experimental task

**DOI:** 10.3389/fpsyg.2026.1885949

**Published:** 2026-08-31

**Authors:** Jade Lanfranchi, Amandine Dervy, Nathalie Angeard

**Affiliations:** 1 Laboratoire Mémoire, Cerveau et Cognition (UR 7536), Université Paris Cité, Paris, France; 2 Institut de psychologie, Université Paris Cité, Paris, France

**Keywords:** assessment, development, empathy, flexibility, neurotypical children

## Abstract

Empathy is conceptualized as a multidimensional construct encompassing affective and cognitive components, yet their developmental trajectories and underlying mechanisms remain incompletely understood. The present research investigated how these dimensions develop across childhood and examined the potential contribution of executive functions, particularly switching abilities, to empathic processing using EmpaDEV1, a novel computerized performance-based paradigm. To address these objectives, two complementary studies were conducted. Study 1 examined age-related cross-sectional changes in affective and cognitive empathy-related processes with varying flexibility demands in a sample of 90 participants distributed across five age groups spanning developmental periods from early childhood to young adulthood (3–20 years). Study 2 introduced a short-improved version of the task (EmpaDEV2) in a sample of 29 children distributed across three age groups (3–12 years), controlling baseline motor speed and key-response learning time, refining the measurement of empathic processing efficiency. Across studies, affective empathy appeared relatively stable across development, whereas cognitive empathy showed significant improvements in both accuracy and response speed with age. Reaction time analyses indicated that cognitive empathy initially involves greater processing demands but becomes increasingly efficient across development. Results showed that increased flexibility demands significantly reduced empathy accuracy across development. Regression analyses suggested that standardized switching task significantly predicted individual differences in cognitive empathy beyond chronological age. Working-memory cueing significantly enhanced performance during early childhood. Convergent validity analyses however revealed moderate associations between EmpaDEV performance and standardized empathy questionnaires. Overall, the findings support an age-related account in which affective and cognitive empathy are initially differentiated and progressively integrated across childhood.

## Introduction

Social cognition encompasses a sophisticated array of perceptual, affective, representational, and regulatory processes supporting interpersonal understanding and adaptive social interaction (Bulgarelli and Jones, 2023; Happé and Frith, 2014). Within this domain, empathy is considered a cornerstone construct, uniquely bridging affective resonance with high-level representational understanding of others' internal states (Decety and Cowell, 2014; Keysers and Gazzola, 2007; Spinrad and Eisenberg, 2014; Zaki, 2014). Despite its centrality, empathy remains conceptually heterogeneous, limiting comparability across studies (Cuff et al., 2014). Nevertheless, converging developmental and clinical evidence identifies empathy as a major determinant of adaptive social functioning and mental health, with impairments implicated in several neurodevelopmental and psychiatric conditions (Farrow and Woodruff, 2007; Metternich et al., 2022; Pastorino et al., 2021; Shalev and Uzefovsky, 2020; Shalev et al., 2025).

Contemporary theoretical frameworks define empathy as a multidimensional phenomenon comprising three primary interacting components: affective empathy (bottom-up emotional arousal and contagion), cognitive empathy (top-down perspective-taking and mentalizing), and empathic concern (the motivational drive toward prosocial behavior; Bulgarelli, 2023; Dvash and Shamay-Tsoory, 2014; Nader-Grosbois, 2023; Sesso et al., 2021; Singer and Lamm, 2009). Developmental work on early prosociality situates empathy within a broader emotion–motivational system (Spinrad et al., 2022). From toddlerhood, children's sensitivity to others' states is expressed not only through emotional understanding, but also through other-oriented actions such as helping, comforting, and sharing, which follow partly distinct developmental pathways (Brownell, 2013; Dunfield, 2014; Paulus, 2014; Warneken and Tomasello, 2006). Other-oriented concern may therefore emerge before fully reflective perspective-taking and become progressively shaped by social experience and the maturation of self-regulatory capacities (Davidov et al., 2013; Malti and Dys, 2018). Affective and cognitive empathy are central to this broader system because they support the detection, sharing, and interpretation of others' emotional states, thereby providing key developmental mechanisms through which other-oriented concern and prosocial responding can emerge. Neuroimaging research has similarly identified partially dissociable neural circuits supporting these dimensions (Decety, 2005; Shamay-Tsoory et al., 2009), with affective resonance typically recruiting limbic and paralimbic structures such as the anterior insula and anterior cingulate cortex, while cognitive empathy engages dorsal and medial prefrontal regions along with the temporoparietal junction (Abu-Akel and Shamay-Tsoory, 2011; Frith and Frith, 2006; Shamay-Tsoory, 2011; Singer et al., 2004). These distinct biological signatures provide the foundation for understanding how empathy components follow unique developmental timetables and exhibit differential sensitivity to environmental, neurobiological, and social influences. However, the boundaries between empathy and related social-cognitive constructs remain debated. Neuroimaging evidence suggests that empathy and Theory of Mind constitute partially overlapping umbrella constructs organized around multiple hierarchically related processes rather than fully separable systems (Schurz et al., 2021). Recent taxonomic proposals have sought to clarify these conceptual boundaries. The Hierarchical Interdependent Taxonomy of Social cognition (HITS) model adopts a narrower definition, distinguishing empathy, the experience of another person's affective state while maintaining self–other distinction, from mentalizing, which encompasses the attribution of mental states, including emotions, through perspective-taking and Theory of Mind (Eikelboom et al., 2025; Quesque et al., 2024). Within this framework, what is commonly termed cognitive empathy largely falls under mentalizing, whereas emotion perception is considered a lower-level process that may contribute to, but is not equivalent to, affective empathy. However, HITS was not designed as a developmental model and does not address how these interdependent processes emerge, differentiate or become coordinated across childhood. Consequently, developmental research may continue to employ the terms affective empathy and cognitive empathy as operational labels, provided that they are understood as referring to overlapping, interacting components of socio-emotional functioning rather than to discrete or process-pure constructs.

## Developmental trajectories and neural specialization

The ontogeny of empathy begins in early infancy through forms of emotional contagion and somatic resonance (Bulgarelli and Jones, 2023; Geangu, 2015; Roth-Hanania et al., 2011). Empirical evidence indicates that infants as young as 8–12 months exhibit rudimentary empathic concern, manifesting as distinctive behavioral and physiological responses to others' distress (Bulgarelli, 2023; Roth-Hanania et al., 2011; Spinrad and Eisenberg, 2014). During toddlerhood, these responses gradually transition into more differentiated empathic reactions as self-awareness and self–other differentiation emerge (Decety and Holvoet, 2021a,b; Sesso et al., 2021; Spinrad and Eisenberg, 2014).

In the preschool period (ages 3–6), recent research by Simon and Nader-Grosbois (2023); Simon and Nader-Grosbois (2025) demonstrates that while empathy dimensions are already differentiated in young children, they exhibit relative longitudinal stability; for instance, each empathy dimension (affective, cognitive, and behavioral) predicts itself one year later, though they do not necessarily cross-predict each other during this stage (Simon and Nader-Grosbois, 2023). Furthermore, the consolidation of a representational Theory of Mind (ToM) between ages 3 and 5 allows for a significant leap in cognitive empathy (Decety et al., 2018). Recent evidence confirms that preschooler profiles can be distinguished by their combined strengths or weaknesses across ToM and empathy dimensions, with girls often showing higher affective empathy on performance-based tasks, even when parent reports suggest no significant gender differences (Simon and Nader-Grosbois, 2023; Simon et al., 2025). These findings suggest that early affective resonance scaffolds later cognitive perspective-taking.

Across middle childhood and adolescence, empathic responding continue to refine alongside increasing specialization of sociocognitive networks (Abu-Akel and Shamay-Tsoory, 2011; Cheng et al., 2014; Overgaauw et al., 2017). Longitudinal work demonstrates that while affective sharing remains relatively stable, cognitive empathy exhibits protracted development well into late adolescence, paralleling the structural maturation of frontotemporal regions (Blaye, 2021; Ma et al., 2025; Sesso et al., 2021). Adolescence represents a sensitive window where empathy becomes increasingly integrated with moral reasoning, social evaluation, and complex identity formation, modulated by neuroplasticity and social experience (Decety and Cowell, 2014; Ganesan and Steinbeis, 2022; Spinrad and Eisenberg, 2014; Zhou et al., 2002).

## Executive functioning as scaffolding for empathic development

Within this broader developmental framework, executive functions, including inhibitory control, working memory, and cognitive flexibility (Diamond, 2013; Horowitz-Kraus et al., 2023; Mairon et al., 2023; Yan et al., 2020; Yetim et al., 2024), are thought to provide regulatory and representational support for empathic processing, particularly in situations requiring children to coordinate their own perspective with another person's emotional state. Theoretical models increasingly position EFs as the necessary “scaffolding” for mature social cognition. Specifically, the emergence of Theory of Mind and cognitive perspective-taking in the preschool period has been shown to depend heavily on the ability to inhibit an egocentric perspective and maintain others' mental states in working memory (Bensalah et al., 2016; Van der Meer et al., 2011). While Carlson and colleagues primarily focused on the EF–ToM link, cognitive control is now understood as prerequisite for the cognitive component of empathy, enabling to flexibly switch between self and other-oriented frames (Lewis-Morrarty et al., 2012; Mairon et al., 2023). However, findings regarding the EF–empathy link remain mixed. Meta-analyses report robust associations with cognitive empathy, whereas links with affective empathy are weaker or inconsistent (Mairon et al., 2023; Yan et al., 2020), likely due to methodological heterogeneity. First, assessment type plays a critical role: studies using performance-based tasks (direct cognitive measures) often yield stronger associations than those relying on parent- or self-report questionnaires, which may be more susceptible to social desirability or limited internal insight (Simon and Nader-Grosbois, 2023; Yan et al., 2020). Second, social-cognitive complexity also matters; tasks involving multiple or incongruent mental states place greater demands on executive resources, particularly flexibility and updating, than tasks assessing a single mental state (Menu et al., 2024; Ruffini et al., 2024). Recent evidence suggests that this cognitive scaffolding remains a transdiagnostic core of socioemotional development. A meta-analysis of 180 studies by Sadozai et al. (2024) identified EF delays as a pervasive feature of neurodevelopmental conditions, suggesting that difficulties in regulating empathic responses may arise from disruptions in top-down control mechanisms (Winters et al., 2025). Similarly, clinical research indicates that children with intellectual disabilities exhibit tightly coupled difficulties in emotion regulation and empathy, reinforcing the view that executive maturation facilitates the online regulation of affective resonance (Simon and Nader-Grosbois, 2025).

## Assessing empathy in children: rationale for research

Despite substantial theoretical progress, variability in findings across studies underscores the need for more integrative assessment paradigms. Inconsistent estimates of associations between EF and empathy highlight the importance of distinguishing between empathy conceptualized as a global trait and as a multidimensional construct. The developmental differentiation of EF structures across childhood must be considered to accurately characterize how these processes support social behavior (Karr et al., 2022; Yangüez et al., 2025). Furthermore, the developmental shift from inhibition-centered networks in childhood to updating-centered networks in early adolescence (Menu et al., 2024) may help explain why different EF components differentially predict specific dimensions of empathy at different ages.

These considerations underscore the need for experimental tools capable of assessing empathy dimensions across development while systematically manipulating executive and contextual demands. The present study addresses these gaps by examining the nuanced interplay between EF and empathy within a developmental framework, moving beyond global associations to identify the specific cognitive mechanisms underlying affective and cognitive dimensions.

## The current study

The present study aims to develop a new computerized experimental task, EmpaDEV1, designed to examine affective and cognitive empathy-related processes across a broad developmental range (3–20 years). EmpaDEV operationalizes two routes to attributing another person's emotional state. In the affective condition, this attribution is supported by visible facial-affective cues embedded in a social context; in the cognitive condition, it must be inferred from the relation between another person's preferences and outcomes when facial cues are unavailable. We retain the conventional labels affective and cognitive empathy for these conditions, while recognizing that the former necessarily recruits emotion perception and the latter desire-based mentalizing and perspective-taking. Emotional contagion is examined separately as a complementary index of affective resonance, whereas empathic concern is not assessed. EmpaDEV should therefore be understood as a performance-based empathy-related socio-emotional inference paradigm rather than as a comprehensive measure of empathy.

Four main objectives are addressed.

First, we examine the age-related trajectories of affective and cognitive empathy (objective 1).

*H1*: Empathy-related skills should improve with age while following distinct age-related trajectories, such that affective empathy stabilizes earlier, whereas cognitive empathy should exhibits a more protracted course, in line with the maturation of executive functions.

Second, we examine the age-related coupling between affective and cognitive empathy (objective 2).

*H2*: These two dimensions should become increasingly independent with age, reflecting a process of developmental specialization.

Third, we investigate the contribution of executive functions to empathy across development by considering both situational and individual sources of variability (objective 3).

*H3a:* At the task level, cognitive flexibility demands are experimentally manipulated. Higher flexibility demands should increase cognitive load and, consequently, reduce empathy performance.

*H3b:* The role of working memory is further examined through a task-support manipulation. Reducing working memory load is expected to facilitate empathy performance, particularly in younger participants.

*H3c:* Executive functioning will also be assessed using standardized tasks. Among executive function indices, switching should be the strongest predictor of empathy performance, with a greater influence in older age-groups.

Finally, we assess the convergent validity of EmpaDEV1 by examining its association with standardized questionnaire measures of empathy (objective 4).

*H4:* Task-based measures should be positively associated with questionnaire-based indices, supporting the validity of the instrument.

## Study 1

### Method

#### Participants

Participants were recruited from schools, leisure centers, and universities in the Île-de-France region. A total of 90 neurotypical participants aged between 3 and 20 years took part in the study (M = 9.72 years, SD = 4.73; Table 1). The sample was divided into five age groups corresponding to key stages of development: 3–4 years (*n* = 13, 14.4%), 5–6 years (*n* = 17, 18.9%), 7–9 years (*n* = 22, 24.4%), 10–12 years (*n* = 21, 23.3%), and 18–20 years (*n* = 17, 18.9%). Age groups were defined to capture major transitions in sociocognitive and executive development, from early theory of mind emergence to mature socio-emotional functioning in adulthood (Blakemore and Choudhury, 2006; Casey et al., 2008; Diamond, 2013; Wellman, 2014). Overall, the sample showed a relatively balanced gender distribution with 42 boys (46.7%) and 48 girls (53.3%).

**Table 1:** Demographic characteristics of the sample (*N* = 90).

Variable	Total sample	Group 1 (3–4y)	Group 2 (5–6y)	Group 3 (7–9y)	Group 4 (10–12y)	Group 5 (18–20y)
Age (years), *M* (SD)	9.62 (4.96)	4.20 (0.28)	5.78 (0.44)	8.15 (0.68)	11.10 (0.71)	19.88 (0.62)
Girls, *n* (%)	48 (53.3%)	9	9	10	10	10

Exclusion criteria included any neurodevelopmental disorder, neurological, or psychiatric history as well as any difficulties that could interfere with task engagement (e.g., attentional, behavioral, or comprehension impairments). Eligibility was assessed through a structured pre-participation interview conducted with parents for minors, or directly with adult participants. This interview screened for neurological or psychiatric history, physical ability to complete the protocol (e.g., ability to sit for approximately 30 min and use a keyboard), languages spoken at home, comprehension of study procedures, and proper completion of consent documentation.

### Measures

#### Computerized Experimental Empathy Task (EmpaDEV1)

A computerized behavioral empathy task was designed using the OpenSesame software with the OSWeb extension (Mathôt et al., 2012) and hosted on the secure JATOS-MindProbe server (Lange et al., 2015). The task was administered on a 12.5-inch Wide Quad High Definition screen (2560 × 1440 pixels). Participants responded using a standard AZERTY keyboard with three adapted child-friendly keys as response buttons, displaying either happy, sad, and neutral smiley faces, designed to be easily identifiable and usable by children as young as 3 years old. The software recorded both response accuracy (correct vs. incorrect) and reaction times (in milliseconds) as complementary dependent variables. Accuracy was considered the primary index of whether participants correctly identified the protagonist's emotional state. Reaction time was included as a secondary index of processing efficiency, rather than as a stand-alone measure of empathy. This distinction is particularly relevant in developmental designs, because age-related changes may be expressed not only through response correctness but also through the speed and efficiency with which children reach a response. In addition, accuracy may approach ceiling in older participants, whereas reaction times can remain sensitive to more subtle developmental changes in task processing. Accordingly, reaction times were interpreted cautiously and always in relation to accuracy patterns.

**Figure 1:**
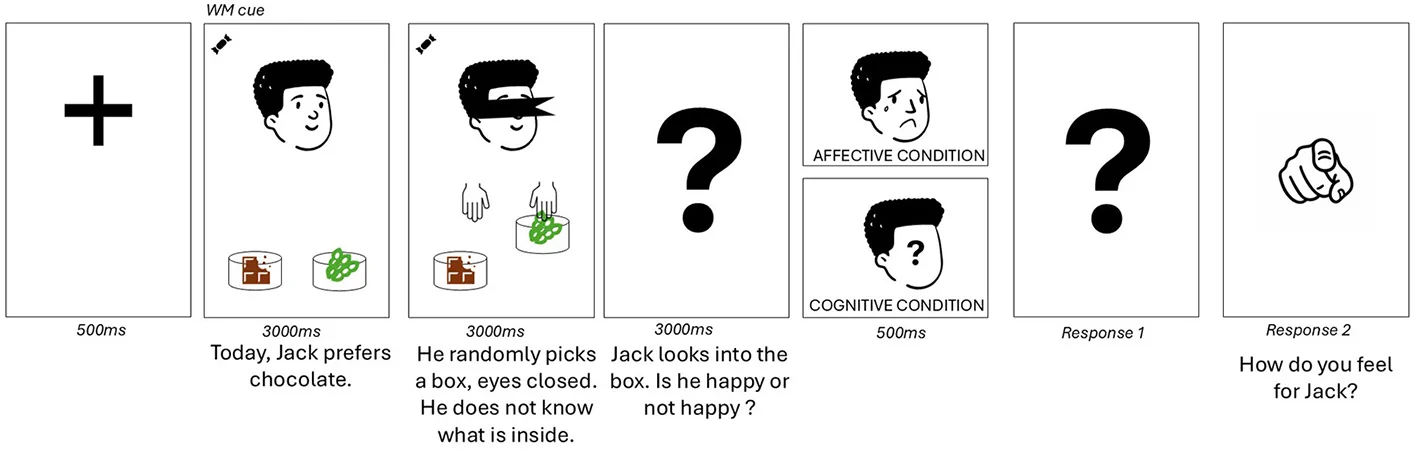
Trial example in the EmpaDEV1 task. WM cue, working-memory cue.

This task was designed to assess affective and cognitive empathy under two experimental conditions manipulating cognitive flexibility (low vs. high) and working-memory load (with vs. without cueing). Participants were presented with short audiovisual stories, each ending with an emotional attribution judgment: they were required to infer the protagonist's emotional state (happy or not happy) and to report their feeling for Jack at the end of the story (Figure 1).

Each trial began with a fixation cross presented for 500 ms. The successive scenes composing the story were then displayed for the duration of the associated audio narration (approximately 3,000 ms per scene). Each trial consisted of a short audiovisual story featuring one of four protagonists (two adults and two children, balanced for gender), selected to examine potential effects of protagonist age and gender.

At the beginning of each story, the protagonist's food preference was explicitly introduced. The protagonist then made a random (blindfolded) choice between two boxes containing foods they either liked or disliked (e.g., chocolate vs. green beans). To manipulate working-memory load, a visual reminder of the protagonist's preference was displayed for half of the participants during the box selection phase (i.e., cue condition, a chocolate icon or a vegetable basket), whereas no reminder was provided in the no-cue condition. After the chosen box was opened and its content revealed, a fixation cross was displayed while the narration promped an evaluation of the protagonist's emotional state. A condition cue (either affective empathy or cognitive empathy) was then displayed for 500 ms, followed by the response screen. Participants were asked to indicate wether the protagonist was happy or not happy, within a maximum response window of 10,000 ms. Responses were recorded using a smiley-based response format and scored as 1 for a correct response and 0 for incorrect responses or “I don't know” answers (neutral face smiley). In the affective empathy condition, the protagonist's face displaying a positive or negative emotional expression (depending on outcome-preference congruence) was shown. In the cognitive empathy condition, the face was replaced by a question mark, requiring participants to infer the emotional state based on the protagonist's preference and the outcome of his choice. Cognitive flexibility demand was manipulated within trials by varying the congruence between the participant's own food preferences and those of the protagonist. Congruent trials were expected to involve lower flexibility demands because the participant and the protagonist shared the same preference, reducing the need for perspective switching. In contrast, incongruent trials required participants to shift from their own preference to the protagonist's conflicting preference when inferring the protagonist's emotional state (e.g., a child who likes chocolate had to infer that a protagonist who likes green beans would be happy to find green beans in the box).

Subsequently, participants reported their own emotional state in response to the question “*How do you feel for Jack?”* Emotional contagion was operationalized as the correspondence between the protagonist's expressed emotion and the participant's self-reported emotion and coded as match, scored as 1, vs. mismatch, scored as 0, across trials (Emotion Contagion Score).

The *EmpaDEV1* task included eight experimental conditions presented in randomized order (see Table 2). Each condition was repeated across the four protagonists (adult woman, adult man, girl, and boy) resulting in a total of 32 trials.

**Table 2:** Experimental conditions crossing empathy type, flexibility demand and emotional outcome valence.

Today, protagonist prefers	*(Flexibility demand)*	Protagonist randomly picks a box, eyes closed. Protagonist picks	Protagonist looks into the box. Is he. she happy or not happy? *(Emotional Valence)*	Emotional expression displayed	*(Empathy condition)*
Chocolate	*Low*	Chocolate	*Happy*	yes	*Affective*
Chocolate	*Low*	Green Beans	*Sad*	yes	*Affective*
Chocolate	*Low*	Chocolate	*Happy*	no	*Cognitive*
Chocolate	*Low*	Green Beans	*Sad*	no	*Cognitive*
Green Beans	*High*	Chocolate	*Happy*	yes	*Affective*
Green Beans	*High*	Green Beans	*Sad*	yes	*Affective*
Green Beans	*High*	Chocolate	*Happy*	no	*Cognitive*
Green Beans	*High*	Green Beans	*Sad*	no	*Cognitive*

The eight conditions described above were repeated for each of the four protagonists. Flexibility Demand was defined as low when the participant's and protagonist's preference were congruent. Independent variables are presented in parenthesis.

### Neuropsychological tasks

#### Language

Language abilities were assessed using the Vocabulary subtests of the WPPSI-IV, WISC-V, or WAIS-IV, depending on participants' age. These subtests evaluate lexical knowledge and verbal reasoning by requiring participants to define orally presented words of increasing difficulty. Age-standardized scores were used in the analyses as an index of verbal ability.

#### Working memory

Working memory was assessed using the forward and backward Digit Span subtests of the WISC-V. In these tasks, participants were required to repeat sequences of digits in the same order (forward span) and in reverse order (backward span), with sequence length increasing progressively. The maximum span achieved in each condition was retained as an index of working memory capacity.

#### Selective attention

Selective attention was measured using the Symbol Search subtests of the WPPSI-IV, WISC-V, or WAIS-IV, depending on age. In this timed visual search task, participants identify whether target symbols are present among distractors. Age-standardized scores were used in the analyses as an index of selective attention and processing speed.

#### Switching

For children, switching ability was assessed using a computerized version of the Hearts and Flowers task (Davidson et al., 2006), adapted for implementation in OpenSesame and deployment *via* JATOS-MindProbe. On each trial, a heart or a flower appeared on the left or right side of the screen. Participants were instructed to press a key on the same side as the stimulus when a heart appeared (congruent trials) and on the opposite side when a flower appeared (incongruent trials). The task comprised three blocks: a congruent block (hearts only), an incongruent block (flowers only), and a mixed block in which both stimuli were presented in random order, requiring flexible switching between response rules. Accuracy scores from the incongruent and mixed blocks were retained as indices of inhibitory control and switching.

For adults, cognitive flexibility was additionally assessed using the Trail Making Test (Parts A and B). In Part A, participants sequentially connect numbered circles, whereas in Part B numbers and letters must be alternatively linked. Completion times and number of errors were retained as indices of processing speed (Part A) and cognitive flexibility (Part B).

#### Diverse desires task

For children aged 3–5 years, understanding of the subjective nature of desires was assessed using the Diverse Desires task (Wellman and Liu, 2004). Children first indicated their own preference between two non-food items (a Rubik's cube and a kite) and were then asked to infer the choice of a protagonist whose preference differed from their own. Performance on this task was used as a control measure of early theory-of-mind abilities.

#### Griffith empathy measure

Trait empathy in children was assessed using the Griffith Empathy Measure (GEM; Dadds et al., 2008; French validation: Girard et al., 2012). This caregiver-report questionnaire evaluates both affective and cognitive components of empathy in everyday situations. The questionnaire was completed by one primary caregiver. Standardized scores were used in the analysis (Dadds et al., 2008; Girard et al., 2012).

#### Interpersonal reactivity index

Trait empathy in adults was assessed using the French version of the Interpersonal Reactivity Index (IRI; Davis, 1980, 1983; French validation: Gilet et al., 2013). This self-report questionnaire measures dispositional empathic tendencies across everyday social situations. Z-standardized scores were computed and used in the analyses (Davis, 1980; Gilet et al., 2013).

### Procedure

All responses were recorded in a single anonymized paper test booklet and subsequently scored. All tasks and activities were administered during a single 30-min testing session, under the supervision of an experimenter trained in the protocol. The assessments were conducted within the host institutions (schools, leisure centers, or universities) in a calm and neutral environment, allowing the proper setup of the equipment and direct interaction between the participant and the experimenter. Tasks were randomly ordered and followed this sequence: (1) the Diverse Desires task (adapted from Wellman and Liu, 2004) for the youngest children in the first two groups, (2) the Symbol Search subtest of the WPPSI-IV/WISC-V (Wechsler, 2012, 2014, 2011), (3) the Vocabulary subtest of the WPPSI-IV/WISC-V/WAIS-IV (Wechsler, 2012, 2014, 2011), (4) an oral digit span forward and backward subtest (maximum span achieved retained), (5) the computerized EmpaDEV1 empathy task, (6a) the Hearts and Flowers task (Davidson et al., 2006), and (6b) the Trail Making Test Parts A and B (Reitan, 1958) for adults. One of the principal caregivers also completed a french translation of (7a) the Griffith Empathy Measure for children (Dadds et al., 2008; Girard et al., 2012) and adults completed (7b) the Interpersonal Reactivity Index (Davis, 1983).

Regarding EmpaDEV1 task conditions, 52.2% of all participants completed the empathy task with a working memory cue to reduce to cognitive load, whereas 47.8% performed the task without. As part of the experimental procedure for manipulating the level of flexibility demand, participants also reported a food preference between green beans and chocolate, with the majority selecting chocolate (90%) over green beans (10%). This preference determined the flexibility level for further analysis, depending on the congruence or incongruence of their own food preference with the empathy task's protagonists'.

### Statistical analysis

An a priori sample size and power calculation was conducted using the effect reported in the princeps study by Van der Meer et al., with an expected between-group difference of 170 ms and an intra-group standard deviation of 290 ms. Using ANOVA framework in R with five groups, α = 0.05, and power = 0.80, the required sample size was estimated at approximately 10 participants per group, corresponding to a total sample of 50 participants. For 95% power, the required sample size was approximately 15 participants per group, corresponding to 75 participants. Although the final age groups were not perfectly balanced, the total sample of Study 1 was 90 participants, exceeding both estimates. Therefore, Study 1 was considered sufficiently powered to detect the expected age-related effect, while remaining less sensitive to smaller interaction or moderation effects.

All analyses were conducted using Jamovi (version 2.3) and IBM SPSS Statistics (version 29) in order to test the corresponding hypotheses for the four main objectives. The collected data were analyzed for outliers and unusual values. All the data were included in the statistical processing. ANOVA/ANCOVA assumptions were inspected before interpretation, and Bonferroni-corrected post hoc tests are reported using adjusted *p*-values.

#### Preliminary analyses

Preliminary analyses were conducted to examine potential confounds and stimulus-related effects before testing the main hypotheses. Factors that did not show robust effects were not retained in the main models in order to preserve model parsimony and interpretability.

First, performance on the Diverse Desires task was examined to ensure that all child participants aged 3–5 years successfully understood the preference-based structure of the paradigm. Responses were scored dichotomously (0 = incorrect, 1 = correct). Children who failed this control task were excluded from subsequent analyses.

Secondly, potential gender differences in global empathy accuracy were assessed using an independent-samples *t*-test to compare girls and boys' performance, using the composite accuracy score across the entire task (affective and cognitive empathy). This was done to determine whether gender should be included as a covariate in subsequent analyses.

Thirdly, the potential influence of the protagonist's characteristics was examined using a mixed-model ANOVA with Age Group (five levels) as a between-subjects factor and Empathy Type (affective vs. cognitive), Flexibility Demand (high vs. low), Protagonist Age (child vs. adult), and Protagonist Gender (male vs. female) as within-subjects factors. These variables were included to control for potential identification effects.

Finally, potential length-related attentional effects were investigated by comparing accuracy between the first and last eight trials using paired-samples *t*-tests and analyzing age-group differences in performance change across the task using a one-way ANOVA on accuracy difference scores.

#### Main analysis

To examine age-related trajectories of affective (AE) and cognitive empathy (CE; Objective 1), mixed-model ANCOVAs were performed with Age Group (between-subject) and Empathy Type (AE vs. CE; within-subject) as factors while controlling for verbal ability, working memory and selective attention. Accuracy and reaction times from the EmpaDEV1 task were used as dependent variables. As expected for a task designed to be accessible across a broad developmental range, adult accuracy was expected to reach ceiling, reducing the sensitivity of accuracy-based analyses; reaction times were therefore considered as a useful complementary index of processing efficiency. Bonferroni-corrected pairwise comparisons were conducted to follow up on significant main effects. We expected a significant main effect of Age Group, reflecting increasing accuracy and decreasing RTs with age, and a significant Age × Empathy Type interaction indicating stronger developmental improvements for CE, whereas AE was expected to reach ceiling earlier.

To investigate the age-related coupling between AE and CE (Objective 2), moderation analyses were conducted using multiple regression models. For both accuracy and reaction times (RTs) in the EmpaDEV1 task, CE performance was predicted from AE performance, centered Age, and the AE × Age interaction term. This approach tested whether the association between AE and CE changes across development, with a significant interaction indicating age-related differentiation between empathy components.

The contribution of executive functions (Objective 3) was examined through complementary analyses.

The impact of task-induced flexibility demands (Objective 3a) was examined using a mixed-model ANCOVA on accuracy scores and correct response times, with Age Group (3–4, 5–6, 7–9, 10–12 years, adults) as a between-subjects factor and Flexibility Demand (low vs. high) and Empathy Type (affective vs. cognitive) as within-subjects factors, controlling for verbal ability, working memory, and selective attention. We predicted a main effect of Flexibility Demand, as well as Flexibility Demand × Empathy Type and Flexibility Demand × Age Group interactions, expecting stronger flexibility-related costs for cognitive empathy and in younger participants, and a possible three-way interaction reflecting possible improvement with age in the differential impact of flexibility demands across empathy components.

Second, the role of switching (Objective 3b) was examined using hierarchical multiple regressions predicting empathy performance. Covariates were entered at Step 1, switching at Step 2, and interaction terms at Step 3. We expected a significant positive contribution of switching, an increase in explained variance (ΔR^2^), and an Age × switching interaction indicating stronger effects with age. Third, the contribution of working-memory load (Objective 3c) was tested using ANOVAs including Cue (cue vs. no-cue) and Age Group. We expected higher accuracy and/or shorter RTs in the cue condition, with stronger benefits in younger participants (Cue × Age interaction).

Finally, to examine the convergent validity of the EmpaDEV1 (Objective 4), Pearson, Spearman, and Kendall correlations were computed between task-based empathy indices (Global Accuracy Score, Mean Reaction Time, AE score, CE score, Emotion Contagion Score) and standardized questionnaire measures (Griffith Empathy Measure and Interpersonal Reactivity Index). We expected small-to-moderate associations in the theoretically expected directions between task-based and questionnaire measures of empathy, reflecting evidence of convergent validity across multimethod assessment.

## Results

Data regarding the initial neuropsychological exploration of the participants' cognitive and executive functioning are presented in Appendix A, alongside standardized measures of empathy in Appendix B.

### Preliminary results

#### Diverse desires task

All participants aged 3–5 years old (*n* = 21) were successful and were therefore included in further analyses.

#### Gender differences

An independent-samples *t*-test was conducted comparing girls and boys on the global empathy accuracy score. The analysis revealed no significant gender differences, *t*(88) = 1.21, *p* = 0.23, Cohen's *d* = 0.25. Although girls showed slightly higher mean accuracy (*M* = 0.89, *SD* = 0.14) than boys (*M* = 0.87, *SD* = 0.16), the difference was small in magnitude and did not reach statistical significance. Empathic performance on the present task (EmpaDEV1) was comparable across genders in this sample and gender was not considered a covariate in subsequent analyses.

#### Protagonist's age and gender

We then examined whether empathy performance varied as a function of the protagonist's age (adult vs. child) and gender (male vs. female) using a generalized mixed-model ANOVA for repeated measures on the global accuracy score of the EmpaDEV1 task.

The main effect of Protagonist Age was not significant, *F*(1, 77) = 0.004, *p* = 0.951, indicating that participants did not differ in their overall performance when responding to adult vs. child protagonists. The interaction between Protagonist Age and Age Group was also not significant, *F*(4, 77) = 0.47, *p* = 0.755, suggesting that the age-related trajectory of performance did not differ as a function of the protagonist's age. No interaction involving participant gender reached significance (largest *F* = 3.25, *p* = 0.076).

The main effect of Protagonist Gender did not reach conventional significance, *F*(1, 77) = 3.55, *p* = 0.063. The interaction between Protagonist Gender and Age Group was not significant, *F*(4, 77) = 0.33, *p* = 0.857. The interaction between Protagonist Gender and participant gender approached significance, *F*(1, 77) = 3.61, *p* = 0.061, but did not reach the conventional threshold. Exploratory post hoc comparisons nevertheless revealed one significant difference: boys showed slightly higher accuracy for male compared to female protagonists (*p* = 0.043, Bonferroni corrected), whereas no other pairwise comparisons were significant. Given the absence of a significant omnibus interaction, this isolated effect should be interpreted cautiously.

The interaction between Protagonist Age and Protagonist Gender was not significant, *F*(1, 77) = 0.60, *p* = 0.442, and no higher-order interactions involving these factors reached significance (all *p*s ≥0.151).

#### Length-related attentional effects

EmpaDEV1 may exhibit a significant length-related effect, reflected in decreased accuracy and/or increased response times as the trial progresses, indicating a decline in attention over the course of the task. As the task was self-paced, cumulative response time was calculated based on the actual presentation sequence in order to capture participants' progression through the task and classify trials as occurring either earlier or later. Cumulative response time was used as it is considered to capture greater variability in performance than error rates alone (Lagattuta et al., 2010). Mean accuracy was computed separately for the first and the last eight trials for each participant. A paired-samples *t*-test was conducted to compare accuracy of early and late trials. Mean accuracy was higher in the first eight trials (*M* = 0.89, *SD* = 0.14) than in the last eight trials (*M* = 0.86, *SD* = 0.21). However, this difference did not reach statistical significance, *t*(df) = 1.45, *p* = 0.151. A one-way ANOVA conducted on the difference score (last eight minus first eight trials) revealed a significant effect of age group, *F*(4, 85) = 5.41, *p* = 0.001, ηp^2^ = 0.20. Younger children showed a marked decrease in performance over time (3–4 years: *M*diff = −0.21; 5–6 years: *M*diff = −0.10), whereas older children and adults showed stable or slightly improved performance (7–9 years: *M*diff = 0.05; 10–12 years: *M*diff = 0.00; 18–20 years: *M*diff = 0.03). Bonferroni post hoc tests indicated that the youngest group (3–4 years) exhibited a significantly larger decline in performance than the 7–9-year-olds (*p* = 0.001), 10–12-year-olds (*p* = 0.015), and adults (*p* = 0.006), while no other group differences reached significance.

## Main results

*Hypothesis 1. Age-related trajectories of affective (AE) and cognitive empathy (CE)*

### Accuracy

To examine the age-related trajectories of affective (AE) and cognitive empathy (CE), a mixed ANCOVA was conducted with Age Group (five levels) as a between-subjects factor and Empathy Type (AE vs. CE) as a within-subjects factor, while controlling for verbal ability (Vocabulaire standard score), working memory (Maximum auditive span backward), and selective attention (Symboles standard score). Box's M test was significant, Box's M = 122.60, F(12, 33257.16) = 9.66, *p* < 0.001, indicating heterogeneity of covariance matrices. Therefore, multivariate statistics based on Pillai's trace were interpreted. The assumption of sphericity was met (Mauchly's *W* = 1.00). Levene's tests were significant for both AE and CE (*p* < 0.001), suggesting heterogeneity of variances across age groups; so the results should be interpreted with caution.

**Figure 2:**
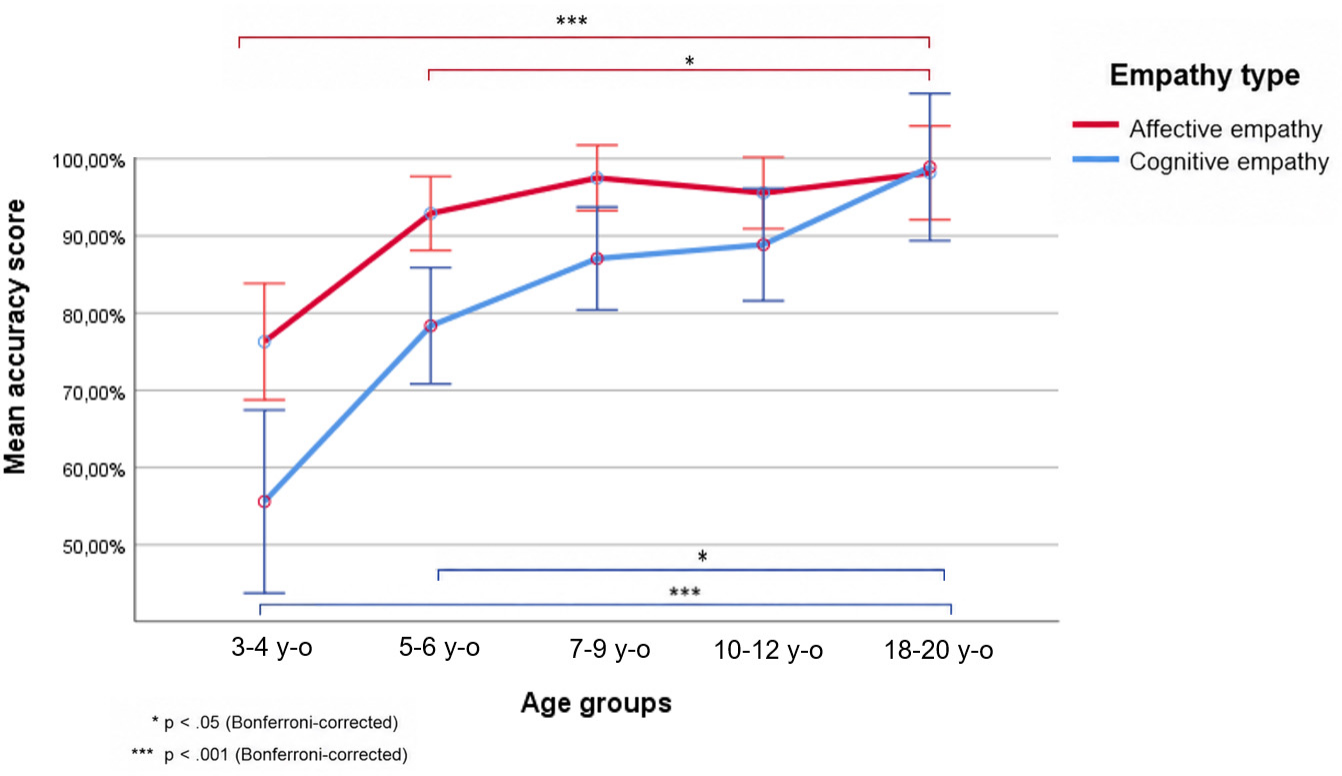
Mean accuracy scores at EmpaDEV1 task in affective and cognitive empathy amongst age groups.

The analysis revealed a significant main effect of Empathy Type, *F*(1, 81) = 5.65, *p* = 0.020, ηp^2^ = 0.065, indicating overall higher accuracy for affective empathy (adjusted *M* = 92.09%, SE = 1.07) than for cognitive empathy (adjusted M = 81.76%, SE = 1.68). Bonferroni-corrected pairwise comparisons confirmed that this difference was significant [mean difference = 10.33%, SE = 1.68, 95% CI (6.99, 13.67), *p* < 0.001]. A robust main effect of Age Group was also observed, *F*(4, 81) = 9.28, *p* < 0.001, ηp^2^ = 0.314, revealing marked improvements amongst age groups in empathy accuracy. Adjusted means increased from 65.94% (SE = 4.01) in the youngest group to 98.54% (SE = 3.23) in adults. Bonferroni-corrected *post hoc* comparisons showed that the youngest group performed significantly worse than all the others (all ps < 0.001). Additionally, Group 2 (5–6 years old) performed significantly worse than adults (*p* = 0.032), though no other pairwise comparisons reached significance (ps ≥ 0.546). This suggests marked developmental improvement followed by a plateau (Figure 2).

Contrary to expectations, the interaction between Age Group and Empathy Type was not significant, *F*(4, 81) = 1.85, *p* = 0.128, ηp^2^ = 0.084, indicating comparable age-related trajectories for affective and cognitive empathy. Nevertheless, adjusted means suggested a gradual convergence with age groups, with the AE–CE gap decreasing from early childhood (AE = 76.30%, CE = 55.59%) to adulthood (AE = 98.17%, CE = 98.91%). Regarding covariates, selective attention was a significant predictor of empathy performance, *F*(1, 81) = 4.29, *p* = 0.041, ηp^2^ = 0.050, whereas verbal ability and working memory were not (ps = 0.241 and 0.796, respectively). Overall, findings indicate robust age-related improvements, consistently higher affective than cognitive empathy accuracy, and parallel developmental trajectories with age for both components.

### Response time

To examine age-related changes in processing speed during affective and cognitive empathy, the same mixed ANCOVA was conducted on correct reaction times. Preliminary assumption checks revealed a violation of the homogeneity of covariance matrices (Box's *M* = 327.30, *F*(12, 33257.16) = 25.80, *p* < 0.001), so multivariate statistics based on Pillai's trace were used instead. The assumption of sphericity was met (Mauchly's *W* = 1.00). Levene's tests indicated heterogeneity of variances for cognitive empathy reaction times (*p* = 0.001) but not for affective empathy reaction times (*p* = 0.279). Therefore, the results should be interpreted with caution.

**Figure 3:**
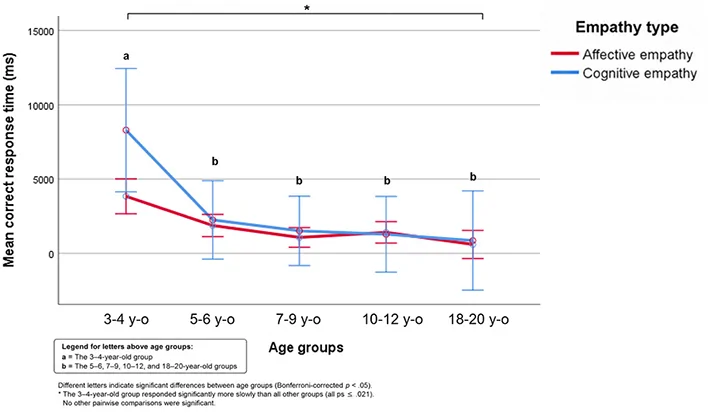
Mean correct response times at EmpaDEV1 task in affective and cognitive empathy condition amongst age groups.

The analysis revealed no significant main effect of Empathy Type, *F*(1, 81) = 0.44, *p* = 0.510, ηp^2^ = 0.005, indicating comparable overall reaction times for affective (adjusted *M* = 1,760 ms, SE = 166.89) and cognitive empathy (adjusted *M* = 2,841 ms, SE = 589.10). Bonferroni-corrected pairwise comparisons confirmed that this difference did not reach significance (*p* = 0.076). A significant main effect of Age Group was observed, *F*(4, 81) = 3.82, *p* = 0.007, ηp^2^ = 0.159, indicating marked improvements amongst age groups in processing speed (Figure 3). Adjusted mean reaction times decreased from 6,066 ms (SE = 1,102.39) in the youngest group to 733 ms (SE = 886.38) in adults. Bonferroni-corrected *post hoc* comparisons showed that the youngest group responded significantly more slowly than all other age groups (all ps ≤ 0.021), whereas no other pairwise comparisons reached significance (ps = 1.000). This suggests a sharp acceleration in development between early childhood and later stages followed by a stabilization of processing speed. The Age Group × Empathy Type interaction was not significant, *F*(4, 81) = 0.86, *p* = 0.490, ηp^2^ = 0.041, indicating similar trajectories for affective and cognitive empathy reaction times.

Regarding covariates, verbal ability, working memory and selective attention did not significantly predict reaction times (all ps ≥ 0.221).

*Hypothesis 2. Age-related coupling between AE and CE*

To examine whether the coupling between affective and cognitive empathy varied with development, continuous interaction models were estimated with age centered as a predictor of the association between the two indices of empathy (Appendix C).

The overall regression model for accuracy scores, was significant, *F*(3, 86) = 23.26, *p* < 0.001, explaining 44.8% of the variance. Affective empathy was a significant predictor of cognitive empathy, β = 0.88, *t*(86) = 2.79, *p* = 0.006, indicating a robust positive association between the two components across the sample. Age was also a significant positive predictor, β = 0.010, *t*(86) = 2.01, *p* = 0.048, reflecting overall increases amongst age groups in performance. However, the interaction between affective empathy and age was not significant, β = 0.0068, *t*(86) = 0.11, *p* = 0.910, indicating that the strength of the association between affective and cognitive accuracy did not vary across development. Thus, no evidence of developmental independence was observed at the level of performance accuracy.

By contrast, the reaction time (RT) model revealed a different pattern between age groups. The regression model was significant, *F*(3, 86) = 5.86, *p* = 0.001, accounting for 17.0% of the variance (*R*^2^ = 0.170). Age significantly predicted cognitive empathy RTs, β = −337 ms/year (centered), *t*(86)=-2.51, *p* = 0.014, indicating faster processing with increasing age. However, the main effect of affective empathy RTs was not significant, β = 0.11, *t*(86) = 0.27, *p* = 0.792 in contrast to the interaction between affective RTs and age, β = −0.30, *t*(86) = −2.57, *p* =0.012. This negative interaction indicates that the association between affective and cognitive processing speeds decreases with age. In younger participants, longer affective latencies were strongly associated with longer cognitive latencies; however, this coupling progressively weakened with development.

*Hypothesis 3a. Effet of task-induced flexibility demand on empathy*

### Accuracy

The effects of flexibility demands were examined using a mixed-model ANCOVA on accuracy scores. This included Age Group (five levels: 3–4 years, 5–6 years, 7–9 years, 10–12 years, adults) as a between-subjects factor and Flexibility Demand (low vs. high) and Empathy Type (affective vs. cognitive) as within-subject factors, Verbal ability, working memory, and selective attention were controlled for. Type III sums of squares were used throughout.

The main effect of Flexibility Demand was significant, *F*(1, 78) = 4.04, *p* = 0.048, ηp^2^ = 0.049, indicating that increasing task-level flexibility demands reduced overall empathy accuracy across participants and conditions. Contrary to predictions, flexibility demands did not differentially influence affective and cognitive empathy. The Flexibility Demand × Empathy Type interaction was not significant, *F*(1, 78) = 1.25, *p* = 0.268, ηp^2^ = 0.016. Furthermore, flexibility demands also did not vary across development, as indicated by the non-significant Flexibility Demand × Age Group interaction, *F*(4, 78) = 0.64, *p* = 0.639, ηp^2^ = 0.032. Finally, the three-way interaction between Flexibility Demand, Empathy Type, and Age Group did not reach statistical significance, *F*(4, 78) = 1.30, *p* = 0.277, ηp^2^ = 0.063.

### Response time

The same mixed-model ANCOVA was used to examine Flexibility effects on reaction times including Age Group (3–4, 5–6, 7–9, 10–12 years, adults) as a between-subjects factor, and flexibility Demand (low vs. high) and Empathy Type (affective vs. cognitive) as within-subject factors, while controlling for verbal ability, working memory, and selective attention. Type III sums of squares were used throughout.

Contrary to predictions, the main effect of Flexibility Demand was not significant, *F*(1, 78) = 0.53, *p* = 0.467, ηp^2^ = 0.007, indicating that increasing task-level flexibility demands did not significantly affect overall reaction times. Importantly, flexibility demands did not influence affective and cognitive empathy differently, as indicated by the non-significant Flexibility Demand × Empathy Type interaction, *F*(1, 78) = 0.79, *p* = 0.376, ηp^2^ = 0.010. Furthermore, flexibility demands did not vary across development, *F*(4, 78) = 1.17, *p* = 0.333, ηp^2^ = 0.056, and the three-way interaction between Flexibility Demand, Empathy Type, and Age Group was not significant, *F*(4, 78) = 1.37, *p* = 0.254, ηp^2^ = 0.065.

*Hypothesis 3b. Increasing contribution of switching abilities to cognitive empathy abilities with age*

To examine the contribution of switching abilities to cognitive empathy performance (Objective 3b), a hierarchical multiple regression analysis was conducted to identify the best predictors of cognitive empathy accuracy. In Step 1, Age (in years), vocabulary (standard score), selective attention (Symbols standard score), and working memory (backward digit span) were entered as covariates. Individual switching abilities (accuracy in the mixed block of the Hearts and Flowers task) were entered in Step 2. The Age × switching interaction term was then entered in Step 3 to test whether the association between switching and cognitive empathy varied throughout development.

#### Step 1—Covariates

The covariate-only model was significant, *F*(4, 84) = 13.80, *p* < 0.001, explaining 39.6% of the variance in cognitive empathy accuracy (adjusted *R*^2^ = 0.367). Age (*b* = 0.01521, SE = 0.00487, *t* = 3.12, *p* = 0.002) and selective attention (*b* = 0.01897, SE = 0.00571, *t* = 3.32, *p* = 0.001) were significant positive predictors, whereas vocabulary (p =0.266) and working memory (*p* = 0.192) were not.

#### Step 2—Addition of switching abilities

Adding switching significantly improved the model, Δ*R*^2^ =0.061, *F* (1, 83) = 9.38, *p* = 0.003, increasing the proportion of variance explained to 45.7% (adjusted *R*^2^ = 0.425). Switching emerged as a significant positive predictor of cognitive empathy accuracy (*b* = 0.384, SE = 0.125, *t* = 3.06, *p* = 0.003). Age (*p* = 0.005) and selective attention (*p* = 0.009) remained significant predictors, whereas vocabulary and working memory remained non-significant (*p* ≥ 0.224).

#### Step 3—Age × switching interaction

The interaction between age and switching did not significantly improve the model fit, Δ*R*^2^ = 0.012, *F*(1, 82) = 1.89, *p* = 0.173). The final model was significant, *F*(6, 82) = 12.10, *p* < 0.001, explaining 47.0% of the variance in cognitive empathy accuracy (adjusted *R*^2^ =0.431). In this final model, switching (*b* = 0.671, SE = 0.243, *t* = 2.76, *p* = 0.007) and selective attention (*b* = 0.01435, SE = 0.00559, *t* = 2.57, *p* = 0.012) remained significant positive predictors. Age showed a non-significant trend (*p* = 0.081), while vocabulary (*p* = 0.280) and working memory (*p* = 0.864) were not significant predictors. The Age × switching interaction was also not significant (*b* = −0.0479, SE = 0.0348, *t* = −1.38, *p* = 0.173).

*Hypothesis 3c. Working-memory cue effect*

We then examined whether the presence of a working memory cue affected global empathy accuracy as a function of age. We conducted a two-way ANOVA was conducted on global empathy accuracy (Empathie_Acc1_TOT) with Age Group (five levels) and Cue condition (with vs. without) as between-subject factors. The results revealed a significant main effect of age, *F*(4, 80) = 18.42, *p* < 0.001, η^2^ = 0.48, indicating substantial age-related differences in performance. There was also a significant main effect of cue *F*(1, 80) = 4.91, *p* = 0.029, η^2^ = 0.06, suggesting that the presence of the cue modestly improved overall accuracy. Importantly, there was a significant between Age × Cue, *F*(4, 80) = 3.12, *p* = 0.019, η^2^ = 0.13. Post hoc comparisons revealed that the cue significantly enhanced performance in the youngest groups (3–4 years and 5–6 years; *ps* < 0.01), whereas no significant benefit was observed for older children (*ps* > 0.10). This pattern suggests that the working memory cue selectively supports empathy performance in early childhood. This is consistent with the idea that executive scaffolding may facilitate the processing of socio-emotional information while cognitive resources are still developing.

*Hypothesis 4. Convergent validity between EmpaDEV1 Task and questionnaire-measures of empathy (GEM, IRI)*

To examine the convergent validity of the EmpaDEV1 task, Pearson, Spearman, and Kendall rank-order correlations were computed between the task-based empathy indices (Global Accuracy Score, Mean Reaction Time, AE score, CE score, Emotion Contagion Score, referring to the proportion of trials in which participants reported an emotional state matching the protagonist's displayed or inferred emotional state, providing an index of emotional resonance) and standardized questionnaire measures (Griffith Empathy Scale, Dadds et al., 2008 and Interpersonal Reactivity Index, Davis, 1980). Descriptive statistics and score ranges are reported in Table 3. EmpaDEV1 scores were available for the entire sample (*N* = 90), whereas Griffith empathy scores were available for the child sub-sample (*n* = 56) and IRI scores for the adult sub-sample (*n* = 16).

**Table 3:** Convergent validity correlations between EmpaDEV1 task scores and questionnaire measures.

#	Variable	Range	1	2	3	4	5	6	7	8	9	10	11	12
1	EmpaDEV Global Accuracy Score	0.28–1.00	—											
2	EmpaDEV Mean Response Time	280–32330	–0.66^***^/ –0.70^***^/ –0.54^***^	—										
3	EmpaDEV Emotion Contagion Score	0–1.00	**0.37** ^***^ **/** **0.44** ^***^ **/** **0.34** ^***^	–0.29^**^/ –0.45^***^/ –0.31^***^	—									
4	EmpaDEV Affective Empathy Accuracy Score	0.31–1.00	**0.85** ^***^ **/** **0.79** ^***^ **/** **0.70** ^***^	–0.65^***^/ –0.58^***^/ –0.47^***^	**0.35** ^***^ **/** **0.38** ^***^ **/** **0.30** ^***^	—								
5	EmpaDEV Cognitive Empathy Accuracy Score	0–1.00	**0.94** ^***^ **/** **0.98** ^***^ **/** **0.92** ^***^	–0.56^***^/ –0.67^***^/ –0.52^***^	**0.33** ^**^ **/** **0.41** ^***^ **/** **0.32** ^***^	**0.63** ^***^ **/** **0.66** ^***^ **/** **0.57** ^***^	—							
6	Griffith_total Z-Score	−3.32 to 0.57	0.19/0.21/0.13	−0.11/−0.24/−0.15	**0.29** ^*^ **/** **0.30** ^*^ **/** **0.21** ^*^	0.13/0.21/0.17	0.18/0.18/0.12	—						
7	Griffith_affective Z-Score	−2.60 to 0.93	0.08/0.16/0.11	−0.00/−0.11/−0.08	0.22/0.14/0.10	0.10/0.15/0.12	0.05/0.15/0.11	**0.85** ^***^ **/** **0.85** ^***^ **/** **0.68** ^***^	—					
8	Griffith_cognitive Z-Score	−4.41 to −0.19	0.01/0.01/−0.01	−0.07/−0.11/−0.06	−0.07/−0.06/−0.03	0.01/−0.03/−0.02	0.01/0.01/-0.00	**0.37** ^**^ **/** **0.35** ^**^ **/** **0.25** ^**^	0.12/0.16/0.12	—				
9	IRI_FantasyScale Z-Score	−2.42 to 1.22	0.34/0.26/0.20	0.10/0.09/0.01	−0.22/−0.28/−0.17	0.50^*^/ **0.4**2/0.36	0.24/0.21/0.16	—	—	—	—			
10	IRI_PerspectiveTaking Z-Score	−3.47 to 1.39	0.45/0.40/0.33	−0.05/0.15/0.12	−0.10/−0.05/−0.03	0.55^*^/0.42/0.36	0.35/0.36/0.29	—	—	—	0.20/0.09/0.08	—		
11	IRI_EmpathicConcern Z-Score	−4.71 to 1.07	**0.50** ^*^ **/** **0.58** ^*^ **/** **0.48** ^*^	−0.21/0.21/0.16	−0.09/0.18/0.16	0.81^***^/0.42/0.36	0.34/0.55^*^/0.45^*^	—	—	—	0.57^*^/0.35/0.29	**0.72** ^**^ **/** **0.69** ^**^ **/** **0.51** ^**^	—	
12	IRI_PersonalDistress Z-Score	−1.74 to 2.44	0.44/0.49/0.40	−0.12/−0.05/−0.03	–0.57^*^/−0.36/−0.24	0.31/0.36/0.31	0.39/0.47/0.37	—	—	—	0.53^*^/ 0.55^*^/ 0.38^*^	0.34/0.47/0.35	0.39/0.41/0.31	**—**

EmpaDEV1intercorrelations: *N* = 90. EmpaDEV1–Griffith correlations (children): *n* = 56. EmpaDEV1–IRI correlations (adults): *n* = 16. Values are presented as Pearson's r / Spearman's ρ / Kendall's τ correlation with ^*^p < 0.05, ^**^p < 0.01, ^***^p < 0.001. Bold values indicate statistically significant correlations (*p* < 0.05).

Across the full sample, strong internal consistency was observed among the EmpaDEV1 indices. Reaction times were negatively associated with all accuracy-based indices (e.g., *r* = −0.66 with Acc1, *p* < 0.001), indicating that faster responses were associated with better task performance. Emotion Contagion scores were moderately associated with Global Accuracy (*r* = 0.37, ρ = 0.44, τ = 0.34, *p* < 0.001), Affective Empathy Accuracy (*r* = 0.35, ρ = 0.38, τ = 0.30, *p* < 0.001) and Cognitive Empathy Accuracy (*r* = 0.33, ρ = 0.41, τ = 0.32, *p* < 0.001).

### Associations between EmpaDEV1 and the Griffith Empathy Measure (children, n = 56)

Correlations between EmpaDEV1 indices and the Griffith Empathy Measures were generally small to moderate (see Table 3). The EmpaDEV1 Emotion Contagion Score showed the most consistent associations, correlating positively with the Griffith total score (*r* = 0.29, ρ = 0.30, τ = 0.21, *p* < 0.05) but not with the Griffith affective or cognitive subscales taken separately (all ps > 0.05). In contrast, global Accuracy, Mean Response Time, Affective Accuracy, and Cognitive Accuracy were not significantly associated with the Griffith total scores or its affective or cognitive subscales. As expected, the total Griffith score and the Griffith affective (*r* = 0.85, ρ = 0.85, τ = 0.68, *p* < 0.001) or cognitive subscale (*r* = 0.37, ρ = 0.35, τ = 0.25, *p* < 0.01) were strongly correlated.

### Associations between EmpaDEV1 and the interpersonal reactivity Index in adults (n = 16)

Several moderate-to-large associations emerged between EmpaDEV1 indices and IRI subscales in the adult subsample. The Global Accuracy Score showed positive correlations with the Empathic Concern subscale of the IRI (*r* = 0.50, ρ = 0.58, τ = 0.48, *p* < 0.05). The Affective Empathy Accuracy Score exhibited the most robust pattern of associations with the questionnaire measures. It showed positive correlations with IRI Fantasy (*r* = 0.50, *p* < 0.05) and Perspective Taking (*r* = 0.55, *p* < 0.05). It also showed a strong positive correlation with IRI Empathic Concern (*r* = 0.81, *p* < 0.001). The Cognitive Empathy Accuracy Score showed a positive association with IRI Empathic Concern at the Spearman and Kendall levels (ρ = 0.55, τ = 0.45, *p* < 0.05), though this did not reach significance at the pearson level. Emotion Contagion scores showed a significant negative association with Personal Distress (*r* = −0.57, *p* < 0.05). The expected intercorrelations were observed within the IRI, including strong associations between Perspective Taking and Empathic Concern (*r* = 0.72, ρ = 0.69, τ = 0.51, *p* < 0.01).

## Discussion—Study 1

The present study examined age-related changes in affective and cognitive empathy throughout childhood using the EmpaDEV1 task and investigated the role of executive functioning, particularly switching, in shaping these abilities. By combining experimental manipulation and neuropsychological measures, the present study aimed to examine cross-sectional age-related differences in empathic performance and to explore whether executive resources are associated with individual differences in affective and cognitive empathy. Overall, the findings provide converging evidence for age-related improvements in empathy, a consistent lag between affective and cognitive dimensions, partial independence of empathic processing dynamics, and support for the idea that cognitive empathy development is moderately influenced by executive functions.

### Age-related improvements in affective and cognitive empathy empathy-related processes

Consistent with our hypothesis, both affective and cognitive empathy improved with age. Children became progressively more accurate and faster at identifying others' emotional states, consistent with progressive maturation of socio-cognitive and regulatory systems (Decety, 2011; Decety and Meyer, 2008; Happé and Frith, 2014). Importantly, however, the task revealed an overall advantage for the affective over the cognitive condition, while both conditions improved with age. Younger children performed more accurately in affective empathy than in cognitive empathy, consistent with the expected developmental asymmetry between the early emergence of affective sharing and the later development of perspective-taking abilities. Affective empathy can be observed from an early age and relies on automatic emotional resonance mechanisms, whereas cognitive empathy depends on representational abilities and executive control which continue to mature throughout childhood (Decety and Jackson, 2004; Shamay-Tsoory, 2011). The persistence of this asymmetry in an experimental task strengthens previous questionnaire-based findings, suggesting that the developmental gap reflects genuine differences in processing demands rather than measurement artifacts (Ruba and Pollak, 2020). Affective responsiveness provides an early scaffold for the subsequent refinement of perspective-taking abilities (Knafo-Noam et al., 2015).

### Coupling and differentiation between affective and cognitive conditions

The second objective was to examine whether empathy dimensions become increasingly differentiated during development. The results revealed a nuanced pattern clarifying how empathic components show age-related changes in efficiency while remaining behaviorally coupled. At the explicit level (accuracy), affective empathy robustly predicted cognitive empathy across the entire age range, and this association remained stable across development. The absence of an Age × Affective interaction suggests that improvements reflect quantitative gains rather than structural reorganization. This stable coupling is consistent with integrative models that propose affective and cognitive processes remain tightly coordinated at the behavioral level (Decety and Cowell, 2014; Heyes, 2018). However, reaction time analyses revealed age-related moderation: the association between affective and cognitive processing speeds decreased with age. Younger children showed strong temporal coupling, suggesting reliance on shared processing resources. As they matured, this coupling weakened, indicating increasing functional independence. This dissociation between stable representational coupling and emerging temporal independence is theoretically informative: reaction-time findings demonstrate a possible age-related differentiation in processing efficiency, suggesting empathy dimensions rely on increasingly distinct cognitive processes with age, even though behavioral performance in both domains remains closely associated across development. Specialization and efficiency of socio-cognitive networks increase across childhood (Blakemore, 2008; Johnson, 2011). Our findings support a model of partial functional independence, in which empathic dimensions become more specialized while remaining coordinated.

### Experimental manipulation of cognitive flexibility demands

Experimental manipulation of cognitive flexibility demands significantly influenced empathy accuracy across participants. More specifically, increasing flexibility demands reduced overall empathic performance independently of age and after controlling for verbal abilities, working memory, and selective attention. However, contrary to our initial hypothesis, this effect did not differ according to empathy dimension, affecting both affective and cognitive empathy. Likewise, no interaction with age group was observed, indicating that the detrimental effect of conflicting perspectives remained relatively stable across development, including in adulthood. Perspective-switching demands may constitute a general cognitive constraint on empathic processing rather than a developmental limitation restricted to younger children or specifically related to cognitive empathy. Successful empathic processing requires the coordination of self- and other-oriented representations, particularly in socially conflicting situations (Apperly, 2012; Decety and Jackson, 2004; Shamay-Tsoory, 2011). Interestingly, flexibility demands affected empathy accuracy but not reaction times. No main effect or interaction involving flexibility demands reached significance for RTs: executive conflict primarily altered the correctness of empathic judgments rather than processing speed itself. This absence of RT effects is coherent with the lack of significant reaction-time differences previously observed between affective and cognitive empathy conditions, and may indicate that flexibility demands interfere more strongly with decisional accuracy than with the temporal dynamics of socio-emotional processing. At the same time, the modest effect sizes observed suggest that short-term experimental manipulations of executive control may only partially capture the complexity of empathic functioning in everyday social situations. Transfer effects from executive manipulations to higher-order socio-cognitive abilities may require more intensive or repeated cognitive engagement to produce substantial behavioral changes (Diamond and Lee, 2011; Diamond and Ling, 2016). Together, these findings highlight the importance of distinguishing between transient state-level executive manipulations and more stable individual executive capacities when examining the relationship between executive functioning and empathy development.

### Individual differences in switching and EmpaDEV1 scores

Individual differences in switching abilities showed a selective and significant association with cognitive empathy performance. Switching remained a significant predictor after controlling for age, vocabulary, working memory, and selective attention, and significantly increased the proportion of explained variance in cognitive empathy accuracy. By contrast, working memory was not a significant predictor in the regression model, suggesting a relatively specific contribution of switching abilities to cognitive empathy performance. Flexible perspective shifting supports the coordination of self- and other-related representations during mental-state reasoning (Carlson and Moses, 2001; Devine and Hughes, 2014). At the same time, executive functioning alone does not appear sufficient to account for developmental improvements in empathy. Although switching abilities contributed uniquely to cognitive empathy performance, age-related developmental changes remained substantial, and the overall pattern of results suggests that broader maturational, social, and experiential factors also play an important role in empathy development (Best and Miller, 2010; Dumontheil, 2016). Rather than supporting accounts positing that executive control constitutes the primary driver of socio-cognitive development, the present findings align with theoretical frameworks proposing that executive functions facilitate the emergence or expression of social-cognitive abilities without fully determining them (Zelazo and Carlson, 2020).

### Working memory cue manipulation

Working memory cue manipulation significantly influenced empathy performance, although this effect varied according to developmental stage. The presence of an external working-memory cue modestly improved global empathy accuracy overall, with this facilitation effect being particularly pronounced in the youngest children (3–4 and 5–6 years old), whereas no significant benefit was observed in older children or adults. Empathic processing in early childhood may rely more heavily on external executive scaffolding when cognitive resources are still immature. From a developmental perspective, maintaining information about another person's preferences or emotional context likely places substantial demands on working memory capacities, particularly during tasks requiring the coordination of self- and other-related representations (Carlson et al., 2004; Diamond, 2013). Experimental work suggested that social-cognitive processes such as perspective-taking require sustained attentional and executive resources, particularly in situations involving competing representations or increased cognitive load (Lin et al., 2010). The present findings converge with models proposing that executive functions, and especially working memory, contribute to the emergence and stabilization of higher-order social-cognitive abilities during childhood (Mairon et al., 2023; Moriguchi, 2014). External cueing may reduce the cognitive load associated with maintaining socially relevant information online, thereby facilitating empathic judgments in younger children whose executive systems remain under development. Early social cognition initially depends on external environmental support before becoming progressively internalized and automatized with age (Vygotsky, 1978; Zelazo, 2015). Interestingly, the effect was restricted to accuracy rather than processing speed, suggesting that working-memory support primarily enhanced the quality or stability of empathic representations rather than the efficiency of processing itself. These results reinforce the idea that executive scaffolding plays a particularly important role during the early developmental stages of empathy.

### Convergent validity of the EmpaDEV1 task

Correlations between task-based empathy scores and questionnaire measures provided partial support for convergent validity. Associations were small to moderate and more consistent for affective empathy, consistent with prior research showing modest convergence between performance-based and questionnaire measures (Dang et al., 2020; Toplak et al., 2013). This modest convergence likely reflects methodological differences between experimental and questionnaire-based assessments. Whereas EmpaDEV1 evaluates empathic performance under controlled conditions, questionnaires primarily capture perceived empathic tendencies in everyday life (Cronbach and Meehl, 1955).

## Rationale for study 2

Study 1 provided initial evidence that EmpaDEV1 was sensitive to age-related changes in empathic processing, with age-related improvements in accuracy and response speed and differential patterns across affective and cognitive empathy conditions. However, these findings also highlighted several methodological constraints that limited the interpretation of some effects, particularly in younger children. In this context, Study 2 was not intended as a full replication of all Study 1 findings, but rather as a targeted methodological refinement designed to strengthen the construct validity, developmental appropriateness, and interpretability of the EmpaDEV paradigm.

Three major limitations were identified. Firstly, children's response times could not be interpreted independently of their motor execution speed because no dedicated motor-time module had been implemented. Second, the task did not include a systematic learning phase for response-key associations, making it difficult to determine whether longer latencies among younger participants reflected genuine empathic processing or uncertainty about the key–emotion mappings. Thirdly, the overall duration of the procedure, involving four distinct protagonists in a 32-trial format, reduced attentional engagement over time, particularly in the final trials. This introduced unwanted variance linked to fatigue or loss of motivation, as well as lack of identification with all of the four protagonists among the youngest participants.

A revised version of the task (EmpaDEV2) was developed. This new version incorporates a motor-time calibration module, enabling individual motor speed to be controlled when interpreting response latencies. It also includes a standardized key-learning module to ensure that any subsequent improvements in performance cannot be attributed to discrepancies in response-key acquisition. Furthermore, the task has been substantially shortened by selecting a single protagonist designed to maximize participant identification. This reduces total testing time and minimizes attentional decline across trials. These additions were also intended to strengthen the interpretation of reaction-time data by reducing potential confounds related to motor execution speed and uncertainty about response-key associations, especially in younger children.

Based on these improvements, the following hypotheses were formulated:

*Hypothesis 1: Contribution of motor execution time*.

We hypothesized that motor execution time would not account for age-related patterns in empathic performance. Although age-related improvements were expected for affective and cognitive empathy, motor time was not expected to significantly predict empathy outcomes once age was controlled for, nor to meaningfully reduce age effects or interact with age. Such findings would suggest that age-related variations in empathy are not driven by motor execution speed.

*Hypothesis 2: Contribution of response-key learning time*.

We hypothesized that response-key learning time would not explain age-related differences in empathic performance. Although age-related effects were expected, learning time was not expected to significantly predict affective or cognitive empathy once age was controlled for, nor to substantially reduce age effects or interact with age. This would suggest that developmental differences in empathy are not attributable to disparities in response-key acquisition.

## Study 2

### Method

#### Participants

Twenty-nine typically developing children participated in the study (*N* = 29). The sample comprised 14 boys and 15 girls. Participants ranged in age from 3.11 to 11.90 years (*M* = 6.53, SD = 2.55). Children were distributed across three age groups reflecting distinct developmental stages: 3–4 years (*n* = 9, *M* = 4.16 years, SD = 0.69, range = 3.11–4.90), 5–6 years (*n* = 10, *M* = 5.60 years, SD = 0.47, range = 5.10–6.30), and 7–12 years (*n* = 10, *M* = 9.59 years, SD = 1.60, range = 7.11–11.90). Participants were recruited from local schools and community networks. Parents provided written informed consent prior to participation, and children gave verbal assent. None of the participants had a reported history of neurological, developmental, or psychiatric disorders.

### Neuropsychological assessment tasks

As in the first study, several subtests from the Wechsler scales (WPPSI-IV or WISC-V, depending on age) were administered to control for cognitive and attentional variables:

- Symbol Search (selective attention)
- Digit Span Backward (working memory)
- Vocabulary (verbal comprehension and language ability)

Executive functioning, particularly motor inhibition and flexibility, was evaluated using the computerized “Hearts and Flowers” task (Davidson et al., 2006).

Finally, the children completed the EmpaDEV2 task, a revised computerized assessment comprising several modules. Each module evaluates a specific component of empathy and related control processes.

#### EmpaDEV2

As in EmpaDEV1, the upgraded version comprises an Affective and Cognitive Empathy Module (Module 3), which constitutes the core component of the task and now involves a single protagonist (a boy) in order to reduce task duration and improve participant identification.

Several additional modules were introduced to better control for non-empathic sources of variability in performance.

*Motor speed module (module 1):* this module was designed to assess baseline motor execution speed and familiarity with the keyboard. The task comprised 10 trials. Each trial began with a fixation cross presented for 500 ms, followed by the appearance of a candy stimulus displayed either on the left or right side of the screen. Children were instructed to “catch the candies” by pressing the corresponding left or right response key as quickly as possible. The response keys were materialized using child-friendly buttons added to the keyboard, similarly to EmpaDEV1. The candy disappeared immediately after a response was provided and was replaced by the next trial. Motor time corresponded to the mean response time across the 10 trials.

*Emotion–key training module (module 2):* this module aimed to ensure that children correctly learned and mastered the association between emotional expressions and response keys before completing the empathy task. Each trial began with a fixation cross presented for 500 ms, followed by the presentation of a drawn child face displaying either a positive or a negative emotional expression. Children were instructed to press the corresponding response key: “happy” when the character appeared happy, “not happy” when the character appeared unhappy, or “I don't know” if they were uncertain. As in EmpaDEV1, the response keys were materialized using child-friendly keyboard buttons displaying a green happy emoji, a red unhappy emoji, and a yellow neutral emoji. The module included 10 trials in total (5 happy faces and 5 unhappy faces). Accuracy scores and reaction times for correct responses were automatically recorded.

### Procedure

The experimental session lasted approximately 30 min and took place individually in a quiet room. Parents first read the information sheet and signed the informed consent form. The study was then explained to the child using age-appropriate language. As in Study 1, the order of task administration was randomized across participants and followed the same general structure. Children first completed (1) the Diverse Desires task (adapted from Wellman and Liu, 2004) for the youngest participants aged 3–6 years, in order to verify their understanding that others may hold preferences different from their own, (2) the Symbol Search subtest of the WPPSI-IV/WISC-V, (3) the Vocabulary subtest of the WPPSI-IV/WISC-V, (4) the Digit Span Forward and Backward subtests, and (5) the computerized Hearts and Flowers task (Davidson et al., 2006). They then completed the EmpaDEV2 task. Within EmpaDEV2, modules were administered in a fixed order. The Motor Speed Module was presented first in order to familiarize children with the response keys and obtain a baseline measure of motor execution speed. The Emotion–Key Training Module was then administered to ensure correct acquisition of the emotional response-key associations. Finally, children completed the Affective and Cognitive Empathy Module (hereafter referred to as Module 3).

### Analysis

All analyses were conducted using Jamovi (version 2.6). Age group (1 = 3–4 years, 2 = 5–6 years, 3 = 7–12 years) was treated as a categorical between-subjects predictor. Study 2 was not designed as a fully powered confirmatory study, but as a preliminary methodological refinement of EmpaDEV2. Given its smaller sample size, the results are interpreted as preliminary and mainly informative regarding task feasibility, motor-speed control, response-key learning, and interpretability.

The total empathy score (Module 3) was used as the primary dependent variable. Parallel analyses of the affective and cognitive subscales yielded comparable patterns. To examine whether motor execution time (Module 1 reaction time) or response-key learning time (Module 2 reaction time) accounted for age-related differences in empathic performance, hierarchical linear regression models were estimated. In each analysis, age group entered the first step to establish the presence of age-groups effects. In the second step, either motor time (Hypothesis 1) or learning time (Hypothesis 2) was added as a covariate. In the third step, the interaction between age group and the respective time variable was entered to test for moderation effects. Model assumptions were examined through visual inspection of residual plots and Q–Q plots, as well as Shapiro–Wilk tests of residual normality. No substantial violations of linearity or homoscedasticity were observed. Residual distributions showed slight deviations from normality, likely attributable to the modest sample size (N = 29). Hovewer, given that linear regression is robust to moderate non-normality in small samples without influential outliers, the analyses were retained as specified. The statistical significance level was set at α = 0.05, and exact *p*-values are reported.

## Results

### Preliminary analyses and age-related differences in empathy

All children successfully completed the Emotion–Key Training Module, achieving the predefined accuracy criterion (> 80%), indicating adequate acquisition of response–emotion mappings. There was a significant age-related improvement in motor speed, *F*(2, 26) = 18.24, *p* < 0.001, justifying its inclusion as a covariate in RT analyses.

A regression model including age group as the sole predictor revealed a significant age effect on total empathy scores, *F*(2, 26) = 7.83, *p* = 0.002, *R*^2^ = 0.376. Older children (7–12 years) demonstrated significantly higher empathy scores than younger children (3–4 years), *p* = 0.002. The difference between the 5–6-year-old group and the youngest group did not reach statistical significance, *p* = 0.118. These findings confirm a robust age-related increase in empathic performance.

*Hypothesis 1: Contribution of Motor Execution Time*

To determine whether motor execution speed accounted for age differences, motor reaction time was incorporated into the regression model. The overall model remained significant, *F*(3, 25) = 6.22, *p* = 0.003, explaining 42.8% of the variance in empathy scores (*R*^2^ = 0.428). However, motor execution time did not significantly predict empathy performance, β = −0.29, *p* = 0.144. The increase in explained variance relative to the age-only model was modest and not statistically significant, Δ*R*^2^ = 0.052, *p* = 0.144. Importantly, the age effect persisted after controlling for motor speed. The contrast between the oldest and youngest groups remained significant, *p* = 0.041, indicating that age differences were not attenuated by the inclusion of motor execution time. Adding the Age × Motor Time interaction did not significantly improve the model fit, Δ*R*^2^ = 0.008, *p* = 0.617, and the interaction term was not significant. Thus, the association between motor speed and empathy did not differ across age groups. Together, these findings suggest that motor execution time does not explain developmental differences in empathic performance, thus supporting Hypothesis 1.

*Hypothesis 2: Contribution of Response-Key Learning Time*

A similar hierarchical regression analysis was conducted including response-key learning time. Again, the age-only model was significant, *F*(2, 26) = 7.83, *p* = 0.002, *R*^2^ = 0.376. When learning time was added to the model, it remained significant, *F*(3, 25) = 6.02, *p* = 0.003. However, learning time was not a significant predictor of empathy scores, β = −0.08, *p* = 0.736, and the change in explained variance was negligible, Δ*R*^2^ = 0.003, *p* = 0.736. The age effect remained significant following the inclusion of learning time, with the oldest group outperforming the youngest group, *p* = 0.010. The Age × Learning Time interaction was also non-significant, Δ*R*^2^ = 0.012, *p* = 0.531. These results suggest that response-key acquisition time does not explain age-related differences in empathic performance, thus supporting Hypothesis 2.

## Discussion

The present research aimed to advance understanding and measurement of empathy development across childhood. Using two complementary studies, we combined the validation of a novel performance-based paradigm (EmpaDEV) with the investigation of developmental and executive contributions to empathic processing. We addressed theoretical and methodological gaps by validating a child-friendly tool assessing affective and cognitive empathy from age three, while clarifying age-related improvements after controlling for confounding factors. Taken together, the findings make four key contributions. First, they confirm robust age improvements in empathic performance across childhood. Secondly, they support a model of persistent integration between affective and cognitive empathy at the behavioral level alongside increasing differentiation in processing efficiency. Third, they demonstrate that age-related gains in empathy cannot be explained by motor speed or response-learning factors. Fourthly, they highlight the specific contribution of executive functions and particularly cognitive flexibility, to cognitive empathy performance across development. Switching abilities remained associated with cognitive empathy after controlling for verbal abilities, working memory, and selective attention, suggesting a specific role of perspective-switching in empathic reasoning.

### Refinement of empathic performance across childhood

Both studies confirmed that empathy continues to develop throughout early and middle childhood with improvements in accuracy and processing speed with age. These results align with developmental models that suggest empathy emerges through the gradual maturation of socio-cognitive and regulatory systems (Decety and Meyer, 2008; Eisenberg et al., 2015). Importantly, Study 2 demonstrated that these gains amongst age groups remained robust even when controlling for motor execution time and response-key learning, strengthening the interpretation that improvements reflect genuine maturation of empathic processing rather than task-related artifacts. This methodological contribution is significant, given that age-related differences in reaction time tasks can be confounded by motor speed and task familiarization (Kail, 2007). By controlling these variables, these controls provide stronger evidence that EmpaDEV primarily captures socio-cognitive maturation. These findings extend classical developmental accounts of empathy by providing experimental evidence on specific performance-based components of empathic processing. Foundational work has emphasized that empathy develops within a broader affective and motivational system involving emotional resonance, empathic concern, sympathy, moral emotions, socialization, and prosocial behavior. The present study complement this broader developmental perspective by isolating two components that can be experimentally manipulated from early childhood onward: the attribution of another person's emotional state when affective cues are available, and the inference of another person's emotional state when the child must integrate preferences and outcomes. In this sense, EmpaDEV provides a controlled way to examine how children become increasingly accurate and efficient in coordinating emotional, contextual, and perspective-related information during empathic judgments.

### Differentiation but cooordination of affective and cognitive empathy-related processes

Findings across studies support a developmental model in which affective and cognitive empathy become increasingly differentiated in processing dynamics while remaining behaviorally integrated. Study 1 demonstrated stable coupling between affective and cognitive empathy in terms of accuracy, suggesting that both components are tightly coordinated in behavioral outcomes. However, reaction time analyses revealed increasing temporal independence between the two components as development progressed, indicating that the underlying processing mechanisms were becoming more specialized. This dissociation between accuracy and reaction-time findings also supports the relevance of considering both indices. Accuracy reflects whether children can correctly identify the protagonist's emotional state, whereas reaction time provides complementary information about the efficiency and potential cognitive cost of this processing. Developmental changes in empathic processing may therefore not be fully captured by accuracy alone, particularly when accuracy approaches ceiling in older children or adults. Study 2 extends this interpretation by demonstrating that age-related improvements persist even when motor and learning factors are controlled for, thus reinforcing the interpretation that changes reflect cognitive rather than low-level processes. Together, the results support a model of functional specialization within an integrated system, consistent with contemporary neurodevelopmental accounts of social cognition (Decety and Cowell, 2014; Johnson, 2011). Rather than strict modular dissociation, empathy development appears to involve increasing efficiency and specialization within a coordinated sociocognitive architecture.

### Executive functions: a supportive role

One of the main objectives of the present research was to clarify the contribution of executive functions to empathy development. Across both studies, the evidence points toward a nuanced conclusion. Study 1 showed that experimentally increasing cognitive flexibility demands modestly reduced empathic performance across participants, independently of age and empathy dimension. Perspective-switching demands may therefore constitute a general cognitive constraint, whereas individual switching abilities contribute more specifically to cognitive empathy. However, performances in the hearts and flowers switching task was associated with cognitive empathy accuracy scores. Cognitive flexibility may make a relatively specific contribution to cognitive empathy development, as switching abilities remained significantly associated with empathic performance even after controlling for working memory, inhibition, selective attention, and verbal abilities. Executive control facilitates the coordination of multiple representations involved in mentalizing (Apperly, 2012; Carlson and Moses, 2001) while emphasizing that the development of empathy depends on broader socio-cognitive processes and social developmental experiences (Best and Miller, 2010). Therefore, executive functioning appears to play a facilitating rather than determining role in empathy development. In line with theoretical accounts distinguishing the role of executive functions in the emergence vs. the expression of socio-cognitive abilities (Zelazo and Carlson, 2020), these findings suggest that executive functions may not constitute the primary developmental basis of empathy itself, but rather support the effective expression of empathic abilities in situations requiring cognitive control, perspective coordination and the updating and maintenance of relevant social information in working memory, along with the management of competing self and other-related representations. Executive inefficiency may therefore limit children's ability to mobilize empathic processes during demanding tasks, particularly for cognitive empathy, without necessarily reflecting reduced empathic understanding.

### Methodological contribution: validating a performance-based empathy-related socio-emotional inference paradigm

This study makes a significant methodological contribution by developing EmpaDEV as a performance-based empathy-related socio-emotional inference paradigm. Importantly, EmpaDEV should not be interpreted as a pure or exhaustive measure of empathy as a broad dispositional construct. Rather, it captures experimentally constrained components of empathic processing, namely children's ability to attribute an emotional state to another person in contexts that vary (1) in the availability of emotional cues, and (2) in the need to integrate another person's preferences and outcomes. In this respect, the affective condition necessarily involves emotion recognition, because the protagonist's facial expression is visible, but this recognition is embedded in a contextualized empathic judgment about another person's emotional state. Conversely, the cognitive condition overlaps with desire-based Theory of Mind, affective perspective-taking, and self–other distinction, because children must infer how the protagonist feels from the relation between preferences and outcomes. This overlap is not considered a limitation of the task *per se*, but rather reflects the fact that cognitive empathy in childhood relies on partially shared socio-cognitive mechanisms. Accordingly, EmpaDEV is best understood as assessing empathy-related socio-emotional inference under controlled conditions, rather than empathy as a global trait. In light of the conceptual distinctions proposed by the HITS model, Those conditions compare two experimentally constrained routes to understanding another person's emotional state. Because HITS was not designed as a developmental model and does not specify the ontogenetic organization of these processes, the present findings contribute to a developmental perspective by examining age-related differences in their efficiency and temporal coordination.

Much developmental literature relies on questionnaires or parent reports, which capture typical behavior but may not fully reflect children's socio-cognitive capacities (Toplak et al., 2013). In contrast, EmpaDEV provides a child-friendly experimental assessment of empathic performance under controlled conditions. Convergent validity findings were limited and should be interpreted cautiously. In children, only the Emotion Contagion Score showed significant associations with the Griffith Empathy Measure, whereas other task-based empathy scores were unrelated to parent-reported empathy dimensions. By contrast, stronger and consistent associations were observed between EmpaDEV and self-reported empathy in adults using the IRI, particularly for affective empathy scores. This discrepancy does not necessarily indicate poor validity, but rather suggests that performance-based tasks, self-reports, and parent reports capture partly distinct levels of empathic functioning. From a multi trait–multi method perspective, convergence is expected to be imperfect when the same construct is assessed through different methods (Campbell and Fiske, 1959). More recent work similarly shows that behavioral tasks and rating scales often correlate only modestly because they assess actual performance under constrained conditions vs. perceived dispositional tendencies in everyday life (Dang et al., 2020; Toplak et al., 2013). This issue may be especially relevant for empathy, as questionnaire-based cognitive empathy is only weakly associated with behavioral empathy performance (Murphy and Lilienfeld, 2019), and parent reports may be influenced by informant-specific perceptions or limited access to children's internal socio-emotional processing (De Los Reyes and Kazdin, 2005). Thus, EmpaDEV should be interpreted as a performance-based measure of contextual socio-emotional inference, with stronger alignment to immediate affective resonance than to broad parent-rated dispositional empathy. Larger validation studies combining parent report, self-report, observational indices, and experimental measures are therefore needed. Study 2 further strengthened the construct validity of the paradigm by showing that the revised version successfully separated empathic processing from motor and learning factors. Together, these studies suggest that EmpaDEV is a promising tool for studying empathy in both typical and atypical children aged three and over.

## Limitations and future directions

Several limitations should be acknowledged. First, both studies relied on relatively modest sample sizes, within each age groups which may have limited the statistical power to detect small executive-function effects on accuracy scores or response time in EmpaDEV1 and 2. Second, the cross-sectional design precludes any direct examination of intra-individual developmental trajectories or developmental reorganization. Findings should therefore be interpreted as age-related differences consistent with a developmental account, rather than as evidence of longitudinal change (Austin et al., 2014; Miller, 2018). Third, although Study 1 included adults as a mature comparison point, the design was primarily focused on childhood. The absence of adolescent groups between late childhood and adulthood limits the interpretation of age-related differences, particularly given the importance of adolescence for executive-function maturation and socio-cognitive refinement. Moreover, because EmpaDEV1 was designed to remain accessible to young children, adult performance may have been affected by reduced task difficulty and motivational engagement. Future studies should therefore include early, middle, and late adolescent groups, ideally within longitudinal designs. Finally, future research should examine additional contributors to empathy development, including social experience and cultural context, and extend EmpaDEV by incorporating more direct behavioral indices of empathic concern, such as spontaneous helping, comforting, or costly prosocial choices. Such developments may strengthen its relevance for research in pediatric clinical populations characterized by socio-cognitive deficits, including neurodevelopmental disorders or acquired brain lesions.

## Conclusion

By integrating preliminary task validation evidence with age-related cross-sectional investigation, the present research provides new evidence that empathic processing becomes more accurate and efficient with age while remaining behaviorally integrated. Age-related improvements cannot be explained by motor speed or task learning and appear only modestly related to executive functioning. These findings support a developmental model in which empathy emerges from the progressive coordination and specialization of socio-cognitive processes within an integrated system.

## Data Availability

The raw data supporting the conclusions of this article will be made available by the authors, without undue reservation.
