# Supplementary material for: The development of cognitive and affective empathy from ages 3 to 20: a new experimental task
Source: Front Psychol. 2026 Aug 31;17:1885949. doi: 10.3389/fpsyg.2026.1885949 (PMC13572134; doi:10.3389/fpsyg.2026.1885949)
Supplement: Supplementary file 1 [file Table_1.docx]

**Appendix A**

*Neuropsychological and standardized empathy measures by age group (M [SD])*

| **Measure** | **3–4y** | **5–6y** | **7–9y** | **10–12y** | **18–20y** |
| --- | --- | --- | --- | --- | --- |
| **Vocabulary (ns)** | 12.15 (2.85) | 12.41 (2.74) | 11.41 (3.03) | 12.67 (2.01) | 10.50 (2.61) |
| **Selective Attention (ns)** | 9.15 (2.48) | 12.29 (3.02) | 13.14 (2.80) | 14.48 (2.60) | 12.18 (4.45) |
| **Digit Span Forward** | 3.27 (0.83) | 4.06 (0.97) | 4.84 (0.61) | 5.14 (1.00) | 6.21 (1.29) |
| **Digit Span Backward** | 1.08 (1.02) | 2.91 (0.87) | 2.95 (0.26) | 3.67 (0.86) | 4.50 (1.16) |
| **H&F Congruent Accuracy** | 0.77 (0.16) | 0.93 (0.16) | 0.98 (0.03) | 0.99 (0.04) | 1.00 (0.01) |
| **H&F Incongruent Accuracy** | 0.64 (0.20) | 0.92 (0.10) | 0.96 (0.06) | 0.96 (0.08) | 0.99 (0.03) |
| **H&F Mixed Accuracy** | 0.60 (0.20) | 0.91 (0.12) | 0.89 (0.14) | 0.94 (0.07) | 0.97 (0.06) |
| **Griffith Total Z** | -1.29 (0.27) | -1.41 (0.81) | -0.93 (1.10) | -0.90 (0.71) | — |
| **Griffith Affective Z** | -0.22 (0.59) | -0.21 (0.53) | -0.15 (0.98) | -0.07 (0.60) | — |
| **Griffith Cognitive Z** | -2.27 (0.69) | -2.12 (1.18) | -2.01 (1.24) | -2.27 (0.99) | — |
| **IRI Fantasy (FS)** | — | — | — | — | -0.41 (1.06) |
| **IRI Perspective Taking (PT)** | — | — | — | — | -0.54 (1.42) |
| **IRI Empathic Concern (EC)** | — | — | — | — | -0.33 (1.45) |
| **IRI Personal Distress (PD)** | — | — | — | — | 0.11 (1.22) |
| **TMTA Time** | — | — | — | — | 20.41 (4.69) |
| **TMTA Errors** | — | — | — | — | 0.18 (0.53) |
| **TMTB Time** | — | — | — | — | 50.29 (12.75) |
| **TMTB Errors** | — | — | — | — | 0.35 (0.49) |
