# Supplementary material for: The development of cognitive and affective empathy from ages 3 to 20: a new experimental task
Source: Front Psychol. 2026 Aug 31;17:1885949. doi: 10.3389/fpsyg.2026.1885949 (PMC13572134; doi:10.3389/fpsyg.2026.1885949)
Supplement: Supplementary file 2 [file Table_2.docx]

**Appendix B**

*Task-Based Empathy Measures*

| **Measure** | **3–4y** | **5–6y** | **7–9y** | **10–12y** | **18–20y** |
| --- | --- | --- | --- | --- | --- |
| **Global Accuracy** | 0.627 (0.205) | 0.857 (0.112) | 0.925 (0.067) | 0.943 (0.084) | 0.971 (0.054) |
| **Global RT (ms)** | 6368.28 (7896.35) | 1985.88 (1142.10) | 1373.75 (631.09) | 1215.15 (1426.08) | 913.75 (525.61) |
| **Affective Accuracy (EA)** | 0.726 (0.214) | 0.926 (0.092) | 0.974 (0.046) | 0.970 (0.061) | 0.989 (0.033) |
| **Affective RT (ms)** | 3765.47 (1514.13) | 1814.59 (1392.13) | 1108.54 (521.64) | 1342.30 (2532.53) | 818.89 (494.67) |
| **Cognitive Accuracy (EC)** | 0.529 (0.284) | 0.787 (0.178) | 0.875 (0.109) | 0.917 (0.130) | 0.952 (0.084) |
| **Cognitive RT (ms)** | 8436.49 (13948.27) | 2157.17 (980.21) | 1638.97 (1056.27) | 1088.01 (825.10) | 1008.61 (688.62) |
| **EA – Low Flex Accuracy** | 0.819 (0.216) | 0.904 (0.083) | 0.977 (0.063) | 0.970 (0.067) | 0.985 (0.061) |
| **EA – Low Flex RT** | 3657.07 (1925.16) | 2238.94 (2567.85) | 1183.05 (872.95) | 838.99 (338.40) | 884.50 (675.85) |
| **EA – High Flex Accuracy** | 0.767 (0.155) | 0.949 (0.125) | 0.972 (0.066) | 0.970 (0.067) | 0.993 (0.030) |
| **EA – High Flex RT** | 3786.81 (1667.54) | 1390.23 (689.76) | 1034.02 (384.20) | 1845.60 (4890.01) | 753.27 (450.62) |
| **EC – Low Flex Accuracy** | 0.592 (0.273) | 0.831 (0.176) | 0.892 (0.124) | 0.929 (0.129) | 0.971 (0.070) |
| **EC – Low Flex RT** | 12812.42 (29814.95) | 2047.68 (798.23) | 1318.41 (928.02) | 1035.05 (1181.29) | 992.44 (964.42) |
| **EC – High Flex Accuracy** | 0.513 (0.322) | 0.743 (0.227) | 0.858 (0.156) | 0.905 (0.147) | 0.934 (0.118) |
| **EC – High Flex RT** | 5404.74 (3412.79) | 2266.65 (1824.48) | 1959.52 (1614.16) | 1140.96 (787.41) | 1024.79 (928.73) |
