# Supplementary material for: The development of cognitive and affective empathy from ages 3 to 20: a new experimental task
Source: Front Psychol. 2026 Aug 31;17:1885949. doi: 10.3389/fpsyg.2026.1885949 (PMC13572134; doi:10.3389/fpsyg.2026.1885949)
Supplement: Supplementary file 3 [file Table_3.docx]

**Appendix C**

*Continuous Interaction Models Predicting Cognitive Empathy From Affective Empathy and Age (N = 90)*

| **Predictor** | **β** | **t** | **p** |
| --- | --- | --- | --- |
| **Accuracy Model** |  |  |  |
| Affective empathy (EA) | 0.88 | 2.79 | **.006** |
| Age (centered) | 0.010 | 2.01 | **.048** |
| EA × Age | 0.0068 | 0.11 | .910 |
| Model fit |  |  |  |
| R² | .448 |  |  |
| F(3, 86) | 23.26 |  | **< .001** |
| **Reaction Time Model** |  |  |  |
| Affective RT (EA_RT) | 0.11 | 0.27 | .792 |
| Age (centered) | −337 | −2.51 | **.014** |
| EA_RT × Age | −0.30 | −2.57 | **.012** |
| Model fit |  |  |  |
| R² | .170 |  |  |
| F(3, 86) | 5.86 |  | .**001** |

***Notes.*** Age was centered prior to computing interaction terms. β values are unstandardized regression coefficients. In the reaction time model, age is expressed in milliseconds per year. Significant effects (*p* < .05) are shown in bold.
